# Social support, oxytocin, and PTSD

**DOI:** 10.3402/ejpt.v5.26513

**Published:** 2014-12-09

**Authors:** Miranda Olff, Saskia B. J. Koch, Laura Nawijn, Jessie L. Frijling, Mirjam Van Zuiden, Dick J. Veltman

**Affiliations:** 1 Department of Psychiatry, Academic Medical Center, University of Amsterdam, Amsterdam, The Netherlands; 2 Arq Psychotrauma Expert Group, Diemen, The Netherlands; 3 Department of Psychiatry, VU University Medical Center, Amsterdam, The Netherlands

**Keywords:** oxytocin, social support, PTSD, trauma, fMRI, neuroimaging, amygdala

## Abstract

**Background:**

A lack of social support and recognition by the environment is one of the most consistent risk factors for posttraumatic stress disorder (PTSD), and PTSD patients will recover faster with proper social support. The oxytocin system has been proposed to underlie beneficial effects of social support as it is implicated in both social bonding behavior and reducing stress responsivity, notably amygdala reactivity (Koch et al., 2014; Olff et al., 2010; Olff, 2012). The amygdala is found to be hypersensitive in people with PTSD.

**Method:**

In order to investigate neurobiological mechanisms underlying potential preventive and therapeutic effects of intranasal oxytocin, we performed a series of fMRI studies (funded with a prestigious NWO TOP grant): BONDS standing for “Boosting Oxytocin after trauma: Neurobiology and the Development of Stress-related psychopathology” in acutely traumatized persons admitted to the emergency department (Frijling et al., 2014); BOOSTER “Boosting oxytocin after trauma: the effects of intranasal oxytocin administration on emotional and motivational processing and neural activity in PTSD” in police officers with and without PTSD.

**Results:**

In this presentation, we present the BOOSTER results on the effects of a single oxytocin administration on amygdala reactivity in response to emotional faces in PTSD patients versus traumatized controls. We found significantly decreased bilateral amygdala reactivity towards emotional faces in PTSD patients compared to traumatized controls.

**Conclusions:**

These promising results call for intervention studies such as studying the effects of medication (oxytocin) enhanced psychotherapy in PTSD patients.
